# Effect of Three Boron Concentrations in Soil on Growth and Physiology in Sweet Cherry Trees

**DOI:** 10.3390/plants12061240

**Published:** 2023-03-09

**Authors:** Gerardo Arredondo, Claudia Bonomelli

**Affiliations:** Departamento de Fruticultura y Enología, Facultad de Agronomía e Ingeniería Forestal, Pontificia Universidad Católica de Chile, Santiago 7820436, Chile

**Keywords:** boron symptoms, sweet cherry biomass partition, photosynthesis

## Abstract

Boron (B) is an essential element for plants. B availability depends on the physical and chemical characteristics of the soil and the quality of irrigation water. Under natural conditions, both toxic and deficit concentrations can occur and should be managed for crop production. However, the range between deficiency and toxicity is narrow. The objective of this study was to determine the response of cherry trees to deficient (0.04 mg kg^−1^), adequate (1.1 mg kg^−1^), and toxic (3.75 mg kg^−1^) B concentrations in the soil by measuring growth, biomass, photosynthetic parameters, visual symptoms, and morphological changes. Plants treated with a toxic dose had more spurs and shorter internodes than those treated with adequate and deficient doses. The white root weight (50.5 g) at low B concentrations had the most roots compared with the adequate (33.0 g) and toxic (22.0 g) concentrations. The stem weight and biomass partitioning were higher for white roots and stems at B-deficient and -adequate doses than at toxic doses. The net photosynthesis (Pn) and transpiration rate (E) were significantly higher in plants with adequate concentrations of B. Stomatal conductance (Gs) was higher in B-deficient plants. Morphological and visual differences were observed between treatments. The results showed that it is essential to adequately manage B in cherry crops to avoid the adverse effects of both low and toxic concentrations.

## 1. Introduction

B is present in all plants, and 80% to 90% is in the cell walls [1]; this and other characteristics make it an essential nutrient [2,3]. After years of research, the role of B in plants and its function in biological processes still raise questions [4]. It is associated with reproductive processes that affect flower development, pollen germination, and pollen tube elongation [5]. Furthermore, owing to its unique properties, B can form complexes with various polyhydroxylated compounds such as sugars and other essential components of plant cell walls [6]. Vascular plants differ in their ability to tolerate excess B, which causes physiological and metabolic changes, and this variability can be explained by differences in the uptake and distribution of B in plants [7].

Boron availability depends on the physical and chemical characteristics of the soil, such as pH, organic carbon content, and the presence of oxidizing molecules. Soil pH is one of the most important factors affecting the availability of B in the soil. Its bioavailability decreases at a higher solution pH [8], which occurs mainly at pH values higher than 6, where B forms insoluble compounds with oxides of iron and aluminum [9]. High organic carbon content increases the availability of B for plants [10]. Owing to changes in rainfall patterns [11], there has been a decrease and increase in the availability of B in the soil, depending on the prevailing conditions [12]. These weather changes have caused alterations in mineral concentrations in the soil. This is essential for B because the limits between deficiency and toxicity are very close [13,14]. Boron toxicity is frequent in arid and semi-arid zones, where soil water evaporation generates micronutrient accumulation in the superficial layer [15]. B toxicity influences the physiological processes associated with photosynthesis and affects the normal development of leaves and roots, which simultaneously decreases plant tolerance to water stress [15]. Symptoms of toxicity in aerial organs include chlorosis, necrosis of the leaf margins, and reduction in plant vigor [15,16]. In the root, excess B affects the growth of meristematic zones, causes the lignification of cortical cells, and results in cellulose accumulation [13]. However, there is differential tolerance between species and cultivars to excessive concentration of this micronutrient, and significant differences were found in root concentration [13,17,18]. On the other hand, boron deficiency is common in highly leached soils, mainly in areas of high rainfall [9]. The common symptoms of B deficiency in leaves are thick, scooped, and brittle leaves [19] as well as holes in the leaves [20].

The cherry (*Prunus avium*) is an economically important export fruit for Chile. It is cultivated from the north-center, where there is naturally a high concentration of B in water and soil [21], to the central zone of the country, where there are no limitations of the element. However, as a result of the decrease in the availability of water in central Chile, new plantations have been displaced toward southern latitudes [22]. In this new area, due to high rainfall and low concentration percentage of organic matter, boron is deficient in these soils [23]. Thus, applying B in this orchard is necessary, considering that the limit between deficiency and toxicity is very narrow [13,14,23]. The aim of this study was to determine the response of cherry trees to B concentrations (deficient, adequate, and toxic) and to document the effects on growth, biomass alterations, physiological responses, visual symptoms, and morphological changes.

## 2. Results and Discussion

### 2.1. Plant Growth Parameters

When comparing the effects of the treatments on the number of aerial organs, a difference was observed in the number of spurs, being higher in plants exposed to toxic doses of boron, followed by the adequate dose, and lowest with a deficient B concentration (Table 1). This is consistent with previous studies on the response to abiotic stress, where the plants accelerate their senescence, contributing to the formation of reproductive structures and redistributing nutrients toward them to ensure the species’ survival [24]. A significantly shorter internode length was also observed at toxic concentrations (3.27 cm) than at adequate (3.77 cm) and deficient (4.03 cm) concentrations. The weight of the white roots showed the same trend in all treatments. Indeed, plants that received a toxic dose of B showed a significant decrease in root weight, in contrast to the findings of Brdar-Jokanović [14], who reported that this type of tissue growth is typical of plants supported for long periods with B deficiency.

In the treatment with a toxic dose of B, which resulted in visible symptoms, the weight of white roots and stems was significantly lower than that of the plants with an adequate and deficient dose, which agrees with the findings of Hua et al. [15], Johnson and Mirza [25], and Riaz et al. [26]. In contrast, Brdar-Jokanović [14] assigned this symptom to B-deficient plants. It has been seen that auxin concentrations in plants that grow under adequate B conditions are low, but they increase strongly with progressive boron deficiency [27]. The increase in auxin concentration could explain the increase in root mass found in plants subjected to B deficiency, as auxins stimulate the growth of lateral roots [28]. The number and weight of leaves, number and weight of buds, trunk length and weight, and brown root weight did not differ significantly between treatments (Table 1).

There were no significant differences in the dry weight (DW) of different plant tissues among the treatments. However, biomass partitioning for white roots and stems showed a significant difference between the treatments. Plants subjected to a low dose of B showed a higher proportion of white-root biomass (12.20) than those with an adequate (8.41) or toxic dose (7.16). Deficient and adequate B concentrations showed higher biomass in stems (1.00) (1.06) than toxic boron concentration (0.6). On the contrary, comparing the treatments, leaves, buds, trunk, and brown roots found no significant differences in the biomass distribution (Table 2).

Destructive sampling and mineral analysis of the tissues were carried out four weeks before leaf fall. The aerial organs, particularly the leaves, were fully developed and mature (old leaves).

Regarding mineral composition, at the toxic B dose, the bud accumulated the highest concentration (640 mg kg^−1^). Gökoğlan et al. [29] demonstrated that in sweet cherry trees, the export of foliage-applied B occurred rapidly, and this B movement was from the leaves to nearby buds, improving B concentration for the next bloom.

Under toxic B conditions, the leaves had a significantly higher concentration (454 mg kg^−1^) than B in the roots (285 mg kg^−1^) and trunk (167 mg kg^−1^), which is consistent with the findings described by Wang et al. [30], who found the same decreasing relationship when they analyzed the same organs in Trifoliate orange. Wang et al. [30] showed a B concentration in old leaves of 418 mg kg^−1^ under a B toxic concentration.

### 2.2. Physiological Parameters

Table 3 shows the effects of deficient, adequate, and toxic concentrations of B in the soil on the photosynthesis parameters of sweet cherry trees. Pn and E were significantly different between treatments. Plants with adequate B concentrations had significantly higher Pn and E than plants with toxic and deficient concentrations. Similar results have been found in citrange oranges and other plants [17,31]. However, Gs did not differ significantly between plants with adequate and toxic B concentrations. This is similar to what was observed in apple trees, in which increasing B concentrations did not affect the intracellular concentration of CO_2_ [32]. Plants with deficient B levels had Gs significantly lower than those with adequate treatments and toxic B concentrations.

### 2.3. Symptoms and Morphological Characteristics

**Figure 1:**
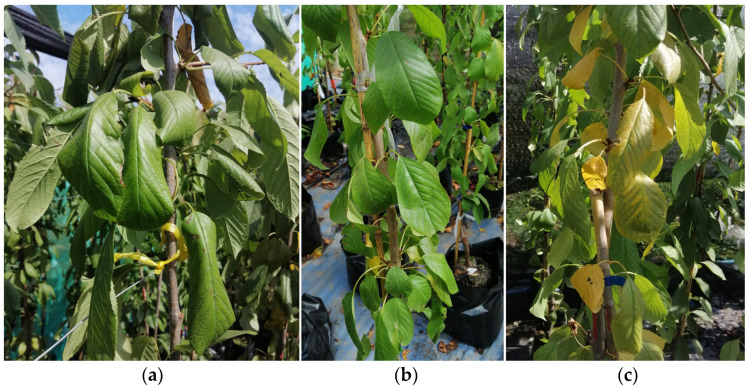
Visual symptoms of sweet cherry leaves with three concentrations of soil boron availability, deficient (**a**), adequate (**b**), and toxic (**c**) (0.04, 1.10, 3.75 mg kg^−1^), respectively.

Symptoms of boron deficiency in cherry leaves included the ruffled shape of the youngest leaves and their thickening (Figure 1a), which were not present at adequate concentrations (Figure 1b) [14,19]. In contrast, mature leaves (old leaves) of cherry plants exposed to toxic boron conditions showed chlorotic and necrotic sectors (Figure 1c). This agrees with the results of Landi et al. [13], García-Sánchez et al. [17], and Wang et al. [30]

**Figure 2:**
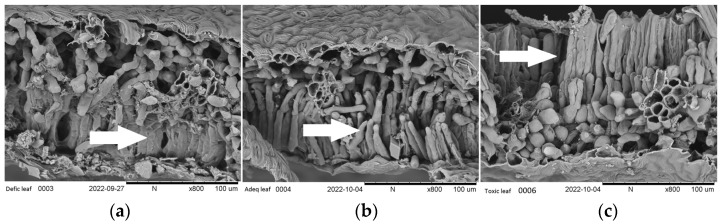
Cross-sectional micrograph of cherry leaves with three concentrations of soil boron availability, deficient (**a**), adequate (**b**), and toxic (**c**) (0.04, 1.10, 3.75 mg kg^−1^), respectively. SEM × 800.

The concentrations of B deficiency and toxicity affected the morphology of leaf tissues (Figure 2), particularly the palisade parenchyma. With an adequate concentration of boron, the leaf cells of this structure appeared turgid (Figure 2b). However, leaves from deficient (Figure 2a) and toxic concentrations have irregular shapes and appear dehydrated, especially in toxic conditions (Figure 2c). The alteration in cell shape could cause lower Pn in the leaves of plants grown under deficient and toxic B conditions. This agrees with observations made in *Arabidopsis thaliana* [33].

**Figure 3:**
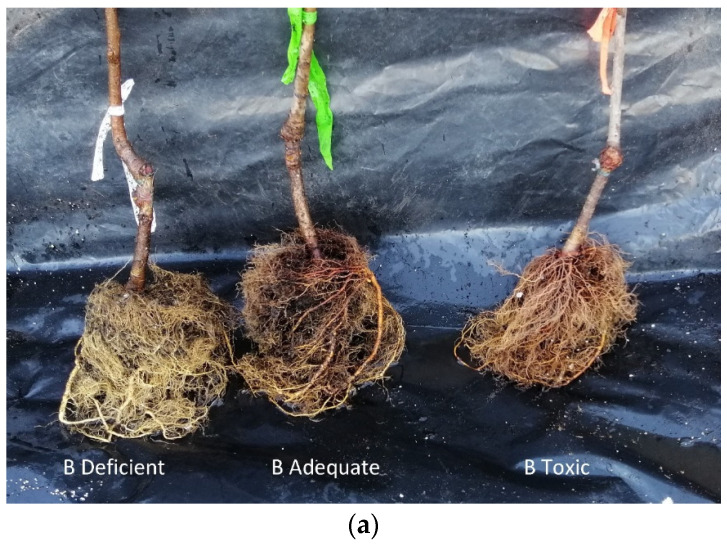

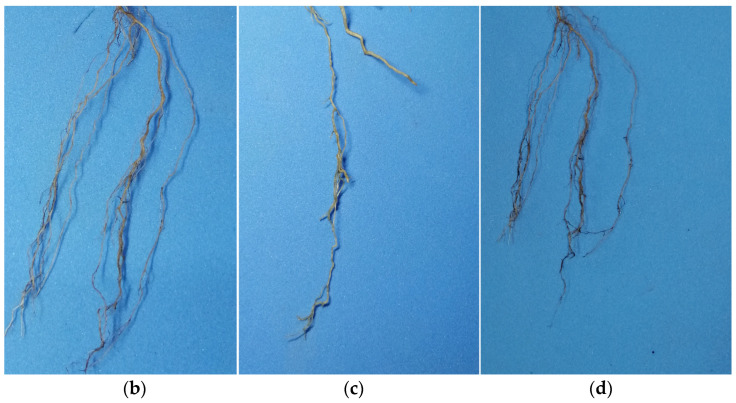
Effect of three concentrations of soil boron availability, deficient (**a**,**b**), adequate (**a**–**c**), and toxic (**a**–**d**) (0.04, 1.10, 3.75 mg kg^−1^), respectively, on the root of cherry trees.

Cherry trees grown in toxic concentrations of B showed low root development and necrosis in the root tips (Figure 3a,d). These results agree with the findings of Kadyampakeni [34]. Excess B in the root affects the growth of meristematic zones, causing lignification of cortical cells and accumulation of cellulose [13]. Plants subjected to a deficient concentration of boron showed a greater root mass volume than plants with adequate and toxic concentrations of B (Figure 3a,b). This is in concordance with the findings of Shireen et al. [19], who reported that plants with deficient nutrient conditions improved their root system and exploratory capacity. Furthermore, it has also been indicated that B deficiency stimulates the formation of secondary roots [27], which would explain this difference. Moreover, depending on age and species, plants have been seen to exhibit a wide range of symptoms [35].

**Figure 4:**
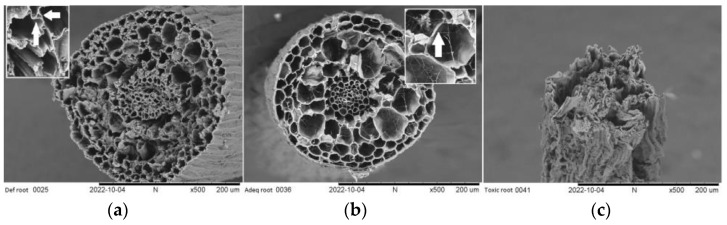
Cross-sectional micrograph of cherry roots with three concentrations of soil boron availability, deficient (**a**), adequate (**b**), and toxic (**c**) (0.04, 1.10, 3.75 mg kg^−1^), respectively, SEM × 500.

Root observations using SEM showed that a lack of structural integrity could be observed in the deficient concentration between the center and xylem vessels when compared with the roots of plants with adequate B levels (Figure 4a vs. Figure 4b). This result agrees with the findings of Koshiba et al. [36]. In addition, it can be observed that under the same conditions of B deficiency, the root cell walls were thicker than in the adequate condition of B (Figure 4a vs. Figure 4b). However, when toxic concentrations occur, the state of tissue deterioration, degradation, and death of the root can be seen (Figure 4c), which agrees with the results of Brdar-Jokanović [14].

## 3. Materials and Methods

### 3.1. Plant Material and Growth Conditions

The experiment was conducted in the Maule region of central Chile (35° 23′ W, 71° 27′ W). The climate is Mediterranean, with mild autumns, cold winters, and hot and dry summers. The experiment was carried out using cherry trees of one-year-old Regina cv. on the Gisela 6 rootstock, placed in 20-L plastic containers, and the soil was composed of 60% washed sand and 40% vermiculite. Trees were drip-irrigated (1 L h^−1^). Calibration was performed, and it was determined that the maximum water retention was 300 mL to avoid water loss by leaching from the soil. The frequency varied depending upon water demand. A basal nutrient solution was used; it contained Ammonium Nitrate (1650.0 mg L^−1^), Calcium Chloride anhydrous (332.2 mg L^−1^), Cobalt Chloride hexahydrate(0.025 mg L^−1^), Cupric Sulfate pentahydrate (0.025 mg L^−1^), Na2EDTA dihydrate (37.26 mg L^−1^), Ferrous Sulfate heptahydrate (27.8 mgL^−1^), Magnesium Sulfate anhydrous (180.7 mgL^−1^), Manganese Sulfate hydrate (16.9 mg L^−1^), Molybdic Acid dihydrate (0.25 mg L^−1^) (sodium salt), Potassium Chloride (0.83 mg L^−1^), Potassium Nitrate (1900.0 mgL^−1^), Potassium Phosphate, monobasic (170.0 mgL^−1^), and Zinc Sulfate heptahydrate (8.6 mg L^−1^), which supplied the nutrients necessary according to the average nutrient requirements of a cherry tree plant during the first year of growth, without boron, and was supplied with 12 L of the basal solution 6 times every 15 days from December to March (southern hemisphere).

### 3.2. Determination of the Experimental B Doses

For the defined deficient and toxic doses, tests were carried out at different concentrations for one year. The low dose was the minimum at which the trees showed growth, and the toxic one was the minimum at which there were visible symptoms. The minimum doses that allowed plant growth (deficient) and showed toxicity symptoms (toxic) were tested for a year. The B source was boric acid.

### 3.3. Experimental Design

The experimental design was completely randomized, with three treatments and three replicates for each treatment, and the whole tree was the experimental unit (total: nine trees). The treatments with B were applied from January to February (every two weeks) with deficient (0.04 mg kg^−1^, adequate (1.10 mg kg^−1^), and toxic (3.75 mg kg^−1^) availability of B.

### 3.4. Soil Mineral Analysis

In the Agroanalysis Laboratory (Universidad Católica de Chile), soil-available B was extracted using hot-water extraction + calcium chloride (CaCl2 0.01 mol L^−1^), and the concentrations were determined with an ICP-Optical Emission Spectroscopy model 5110, Agilent Technologies, Victoria, Australia. The laboratory has a Quality Assurance/Quality Control (QA/QC) program, which ensures that scientifically credible and meaningful data are collected.

### 3.5. Biomass Components and Their Partitioning, DM, and Mineral Composition Measurements

The number and weight of aerial organs and roots were determined after a destructive procedure was performed in March. Each harvested tree was washed, and all leaves, buds, spurs, stems (shoots), trunk, white roots (1.0–2.0 mm in diameter, without lignification), and brown roots (>5.0 mm in diameter with tissues lignification) were separated and measured (Table 1). The separated organs were assessed in an Agroanalysis Laboratory, where the dry matter and mineral composition were determined. The vegetal samples (trunk, stems, spurs, buds, leaves) were taken and oven-dried for 48 h at 65 °C to obtain dry matter content. Then, the tissues were converted to ash by dry combustion at 500 °C, and ashes were dissolved in acid solution (HCl 2 mol L^−1^). The concentrations were determined with an ICP-Optical Emission Spectroscopy model 5110, Agilent Technologies, Victoria, Australia [37].

### 3.6. Physiological Parameters

After applications, the water supply was suspended for two days after the last irrigation. The photosynthetic parameters Pn, Gs, and E were measured simultaneously with those of growth using IRGA CI 340.

### 3.7. Morphological Study

Three root and leaf samples for each treatment were separated for morphological analysis, which was carried out by scanning electron microscopy (SEM) using a Hitachi TM 3000 microscope.

### 3.8. Statistical Analysis and Data Presentation

The data were tested for homogeneity of variance and normality of distribution. The significance was determined by analysis of variance (ANOVA), and the significance (*p* < 0.05) of any differences between mean values was tested by LSD Fisher test, using INFOSTAT (Universidad Nacional de Córdoba, CO, Argentina) [38].

## 4. Conclusions

The minimum soil B concentration (deficient) that allowed the growth of cherry trees was 0.04 mg kg^−1^. The adequate soil B concentration was 1.1 mg kg^−1^, and the soil B concentration at which there were visible toxic symptoms was 3.75 mg kg^−1^. These different doses of B in the soil produced several effects on cherry trees, including growth (higher number of white roots at deficient concentrations, higher spur number, lower stem fresh weight, and lower internode length at toxic concentrations), physiological (higher net photosynthesis and transpiration rate in adequate concentration, and lower stomatal conductance in deficient concentration), and morphological (parenchyma structure alteration) parameters. At toxic B concentrations, buds and old leaves accumulated higher B contents than other organs. This communication shows different approaches to the effects of the three B concentrations in soil on the cherry trees.

## Figures and Tables

**Table 1:** Effect of three concentrations of soil boron availability, deficient, adequate, and toxic (0.04, 1.10, 3.75 mg kg^−1^), respectively, on the plant growth parameters of sweet cherry trees.

Plant Tissues	Treatments (Boron in Soil)			*p* Value
	Deficient	Adequate	Toxic	
Leaves (n°)	132.0 a ^1^	153.0 a	128.0 a	0.960
Buds (n°)	72.0 a	74.0 a	54.0 a	0.346
Spurs (n°)	4.0 c	6.0 b	10.0 a	0.040
Stems (n°)	6.0 a	7.0 a	2.0 a	0.134
Internode length (cm)	4.03 a	3.77 b	3.27 c	0.050
Trunk length (cm)	153.5 a	150.5 a	168.5 a	0.282
Fresh weight of (g)				
Leaves	142.9 a	155.3 a	145.5 a	0.962
Buds	3.15 a	2.07 a	1.27 a	0.320
Stems	4.1 a	4.1 a	2.10 b	0.050
Trunk	206.0 a	202.5 a	201.0 a	0.990
White roots	50.5 a	33.0 b	22.0 c	0.035
Brown roots	94.5 a	94.0 a	93.5 a	0.868

^1^ Different letters in the same row denote a significant difference (*p* ≤ 0.05).

**Table 2:** Effect of three concentrations of soil boron availability, deficient, adequate, and toxic (0.04, 1.10, 3.75 mg kg^−1^), respectively, on biomass partition in sweet cherry trees.

Dry Weight of Plant Tissues (%)	Treatments (Boron in Soil)			*p* Value
	Deficient	Adequate	Toxic	
Leaves	12.97 a ^1^	14.96 a	14.33 a	0.960
Buds	0.75 a	0.52 a	0.34 a	0.346
Stems	1.00 a	1.06 a	0.61 b	0.048
Trunk	50.06 a	51.10 a	53.15 a	0.134
White roots	12.20 a	8.41 b	7.16 c	0.050
Brown roots	23.02 a	23.95 a	24.41 a	0.282

^1^ Different letters in the same row denote a significant difference (*p* ≤ 0.05).

**Table 3:** Effect of three concentrations of soil boron availability, deficient, adequate, and toxic (0.04, 1.10, 3.75 mg kg^−1^), respectively, on photosynthesis parameters of sweet cherry trees.

Physiological Parameters (%)	Treatments (Boron in Soil)			*p* Value
	Deficient	Adequate	Toxic	
Net Photosynthesis (Pn) umol CO_2_ m^−2^ s^−1^	7.98 c ^1^	14.85 a	10.34 b	<0.001
Stomatal conductance (Gs) mmol m^−2^ s^−1^	94.60 b	146.20 a	137.85 a	<0.001
Transpiration rate (E) mmol m^−2^ s^−1^	2.38 b	3.23 a	2.66 b	<0.001

^1^ Different letters in the same row denote a significant difference (*p* ≤ 0.05).

## Data Availability

The data presented in this study are available on request from the corresponding author.
